# Cysteine-Rich Hydrophobin Gene Family: Genome Wide Analysis, Phylogeny and Transcript Profiling in *Cordyceps militaris*

**DOI:** 10.3390/ijms22020643

**Published:** 2021-01-11

**Authors:** Xiao Li, Fen Wang, Yanyan Xu, Guijun Liu, Caihong Dong

**Affiliations:** 1 State Key Laboratory of Mycology, Institute of Microbiology, Chinese Academy of Sciences, Beijing 100101, China; lixmushroom@gmail.com (X.L.); wangfen@im.ac.cn (F.W.); xuyanyan@im.ac.cn (Y.X.); 2 College of Life Science, University of Chinese Academy of Sciences, Beijing 100101, China; 3 Beijing Radiation Center, Beijing 100101, China; liuguijun@brc.ac.cn; 4 Guizhou Key Laboratory of Edible Fungi Breeding, Guizhou Academy of Agricultural Sciences, Guiyang 550000, China

**Keywords:** *Cordyceps militaris*, hydrophobin, lifestyles, fruiting body development, light response, multi-domain hydrophobin

## Abstract

Hydrophobins are a family of small secreted proteins found exclusively in fungi, and they play various roles in the life cycle. In the present study, genome wide analysis and transcript profiling of the hydrophobin family in *Cordyceps militaris*, a well-known edible and medicinal mushroom, were studied. The distribution of hydrophobins in ascomycetes with different lifestyles showed that pathogenic fungi had significantly more hydrophobins than saprotrophic fungi, and class II members accounted for the majority. Phylogenetic analysis of hydrophobin proteins from the species of *Cordyceps* s.l. indicated that there was more variability among the class II members than class I. Only a few hydrophobin-encoding genes evolved by duplication in *Cordyceps* s.l., which was inconsistent with the important role of gene duplication in basidiomycetes. Different transcript patterns of four hydrophobin-encoding genes during the life cycle indicated the possible different functions for each. The transcripts of *Cmhyd2*, *3* and *4* can respond to light and were related with the photoreceptors. CmQHYD, with four hydrophobin II domains, was first found in *C. militaris*, and multi-domain hydrophobins were only distributed in the species of *Cordycipitaceae* and *Clavicipitaceae*. These results could be helpful for further function research of hydrophobins and could provide valuable information for the evolution of hydrophobins.

## 1. Introduction

Hydrophobins are small surface-active proteins produced exclusively by filamentous fungi [1,2]. They are found in different fungal structures, such as aerial hyphae, spores, and fruiting bodies, all of which are hydrophobic and can self-assemble into amphiphilic layers at hydrophilic/hydrophobic or air/water interfaces [3,4,5]. This hydrophobin protein family owns a strictly conserved motif with eight cysteine residues [6], which can form four disulfide bridges to connect the β-strands as well as stabilize the protein structure [7].

According to the spacing between the conserved cysteine residues, distinct hydropathy patterns and physical properties, hydrophobins are traditionally divided into two classes (class I and class II) [8,9]. Class I hydrophobins form highly insoluble membranes in water, organic solvents and 2% SDS (sodium dodecyl sulfate), while the membranes formed by class II hydrophobins can be dissolved in aqueous ethanol (60%) or 2% SDS [3]. Hydrophobins have been reported in the phyla Ascomycota and Basidiomycota filamentous fungi [8,10]. There are only class I hydrophobins in basidiomycetes, whereas both classes have been identified in ascomycetes [6]. A significant expansion of hydrophobin-encoding genes in basidiomycetes was reported (1–40 copies), whereas contraction through gene loss was observed among the analyzed ascomycetes (1–11 copies). Further phylogenetic analysis confirmed the important role of gene duplication events in the evolution of hydrophobins in basidiomycetes [8]. An increased number of hydrophobin-encoding genes appeared to have been linked to the species’ ecological strategy, with the non-pathogenic fungi having increased numbers of hydrophobins compared with their pathogenic counterparts [8] based on the analysis of both basidiomycetes and ascomycetes. However, most of the species of basidiomycetes used with abundant hydrophobins were non-pathogenic fungi, which may affect the correlation analysis.

Hydrophobins are found to play a role in aerial hyphae, mounds (a neoplasm of dikaryotic fruiting body), fruiting body formation and the development of macro-fungi in basidiomycetes [11,12], and in cell wall integrity, conidiation, hydrophobicity, fungal pathogenesis and so on in ascomycetes [13,14,15]. The first characterized hydrophobin was SC3 from *Schizophyllum commune*, which plays an essential role in the formation of aerial mycelium [16]. In *Tricholoma vaccinum*, nine hydrophobin genes were identified and their differential expression in the life cycle revealed the important role of the different hydrophobins for aerial mycelium, fruiting body and ectomycorrhiza establishment [17]. All the ten hydrophobin I members in *Flammulina filiformis* showed relatively higher levels of expression in the primordial stages of the fungus [18], and *Hyd9* was confirmed to play an important role in the aerial hyphae and fruiting body formation by RNAi and overexpression [12]. In mycoparasite fungi *Trichoderma longibrachiatum*, hydrophobins affected the hydrophobicity of conidia, disease resistance, pathogenicity and plant growth promotion activity [14]. In entomopathogenic fungi *Metarhizium brunneum*, hydrophobins participated in the hydrophobicity and pathogenicity of hypha [13].

It was reported that the expressions of some class I and II hydrophobin genes were stimulated by light in some fungi [19,20,21], and this stimulation has been linked to the circadian clock [22]. Ten out of 13 hydrophobin genes were downregulated at least 2-fold in light-grown colonies of blue-light acceptor mutant Δ*wc2*Δ*wc2* dikaryon compared with the wild-type dikaryon in basidiomycetes *S. commune* [20]. Eight out of 10 hydrophobin II genes were upregulated in light dependent on one or both blue-light regulator proteins BLR1 and BLR2 in *T. atroviride* [19,23,24]. In *T. reesei*, hydrophobin-II *hfb2* and *hfb3* were downregulated in mycelia with constant light conditions compared with the darkness [21]. The expression of the class I hydrophobin gene *eas* was controlled by the circadian clock gene [22] and accumulated at mycelia after 180min illumination in *Neurospora crassa* [25].

*Cordyceps militaris,* one of the entomopathogenic fungi, is also a well-known edible and medicinal mushroom. *C. militaris* has been widely used as an herbal drug and tonic in East Asia and has also been studied worldwide owing to its various biological activities, such as anti-inflammatory, anti-tumor [26], anti-influenza virus [27], and radioprotection [28] activities. The fruiting bodies of this fungus have been successfully cultivated and commercialized. It has been listed as a novel food by the Ministry of Health of the People’s Republic of China in 2009.

*C. militaris* is considered as a model organism for the study of over 400 species of *Cordyceps* spp. that has been described [29]. The mechanisms of photo reaction and fruiting body development have been studied by this team for many years [30,31,32,33,34]. During the research, it was found that hydrophobins may play a role in both photo reaction and fruiting body development.

In this study, four hydrophobin-encoding genes were identified from the *C. militaris* genome. They were classified according to the protein domain and hydropathy pattern. The correlation between the number of hydrophobins from the genomes of 47 Ascomycota fungi and their ecological strategy was revealed. Phylogenetic analysis of 90 hydrophobins from 16 species of *Cordyceps* sensu lato was performed. Transcript levels of the hydrophobin genes during the life cycle and dynamic transcript patterns in response to different light exposure times were characterized to infer potential roles for each of the hydrophobins in this fungus. A multi-domain hydrophobin with four hydrophobin II units was found in *C. militaris*, and the multi-domain hydrophobins were only found in the species of *Cordycipitaceae* and *Clavicipitaceae*. These results could provide useful information for further functional investigations of the hydrophobin gene family.

## 2. Results

### 2.1. Domain Structure, Hydropathy Pattern and Homology Modeling of Hydrophobins in Cordyceps militaris

Four hydrophobin genes termed as *Cmhyd1*-*Cmhyd4* were identified in both *C. militaris* genomes (Table 1). *Cmhyd1*, *2* and *3* contained two introns and *Cmhyd4* contained one intron (Figure S1). All identified hydrophobin proteins had N-terminal signal sequences and therefore had the possibility of being secreted (Table 1). They contained approximately 90–150 amino acids with a molecular weight of 10–15 kDa. Alignment of the sequences of four hydrophobin proteins showed conserved cysteine residues necessary for disulfide bridges formation, a characteristic feature of all fungal hydrophobins (Figure S2). They contained 60–90 amino acids core structure displaying the eight cysteines, except CmHYD2, which had only six cysteines. There was no hydrophobic amino acid tryptophan in all the four hydrophobins.

Motif analysis showed that CmHYD1 contained Pfam06766 (hydrophobin 2). It was found to be a class II member which had a short stretch of amino acids (only eight) between cysteine residues C3 and C4. It had a cysteine pattern of CX9-CCX8-CX19-CX8-CCX10-C (where X signifies any other amino acid than cysteine) (Figure S2 and Table S1). There were 11 continuous glycines in the N-terminal of CmHYD1 (Figure S2). Likewise, it was reported that a small number of hydrophobins in genus *Trichoderma* contained an extended N-terminus rich in either proline and aspartate, or glycine-asparagine [35].

CmHYD2 also contained Pfam06766 (hydrophobin 2) and the sequence identity with CmHYD1 was 46.67% with coverage 97% (E-value of 7 × e^−30^). CmHYD2 contained only six cysteines, lacking one cysteine in the two CC doublets, respectively (Figure S2 and Table S1). However, it had the same cysteine spacing pattern with CmHYD1, except that the cysteine was replaced by serine and alanine in the two CC doublets, respectively (Figure S2).

Both CmHYD3 and CmHYD4 contained pfam01185 (Fungal hydrophobin) and they were found to be class I members. There was a long stretch of amino acids (37 and 25, respectively) between the C3/C4 position and the cysteine pattern was displayed as CX7-CCX37 or 25-CX17-CX5-CCX10-C (Figure S2 and Table S1). CmHYD3 and CmHYD4 only had 34.74% sequence identity (coverage of 68% and E-value of 9e^-6^).

The hydropathy profiles were compared between the two class members of hydrophobins (Figure S3A–D). Class I members (CmHYD3 and CmHYD4) showed a higher hydrophobicity stretch (positive values) (Figure S3C,D) than class II members (CmHYD1 and CmHYD2) (Figure S3A,B). In addition, the cysteine doublets in the class II hydrophobins were followed by hydrophobic residues (Figure S3A,B), whereas in the proteins belonging to class I, the cysteine doublets were followed by hydrophilic residues (Figure S3C,D). The hydropathy profiles of class I and II hydrophobins in *C. militaris* were consistent with the typical characteristics of class I and II members [8].

Homology modelling was performed towards establishing the structure of a subset of hydrophobin sequences, revealing the distribution of the hydrophobic residues and conserved cysteine residues (Figure S4A–D). The templates of each hydrophobin for homology modelling were described in Figure S5. This homology modelling clearly showed that the residues were arranged as patches. Each hydrophobin had one or two α-helixes and three to five β-hairpins (Figure S4). The amino acid sequence encoded by the second exon of *Cmhyd1* and *Cmhyd2* folded exactly into the single α-helix of the hydrophobin, just like the hydrophobin genes of *H. jecorina, H. virens* and *H. atroviridis* [35]. The β-hairpins of CmHYD1, CmHYD2 and CmHYD3 conducted into the β-barrel (Figure S4A–C). The hydrophobins contained eight cysteine residues and formed four disulfide bridges, which is a distinctive feature of hydrophobins [4]. The three-dimensional structures showed that four disulfide bridges were symmetrically located in almost the same plane in the hydrophobins’ structure: bridge C1-C6, C2-C5, C3-C4 and C7-C8 in *C. militaris* (Figure S4A,C,D), except that CmHYD2 conducted three disulfide bridges: bridge C1-C6, C2-C5 and C4-C8 (Figure S4B). The first bridge, C1-C6, connected the N-terminal loop to the small barrel formed by β-hairpins, the second bridge, C2-C5, connected the α-helix to the β-barrel in CmHYD1, CmHYD2 and CmHYD3, and the last two bridges, C3-C4 and C7-C8, in CmHYD1, CmHYD3 and CmHYD4 or the last bridge, C4-C8, in CmHYD2 were located inside the β-barrel (Figure S4A–D).

### 2.2. Genomic Organization of the Hydrophobin Genes

The assembly revealed that *C. militaris* had seven chromosomes [36]. The gene *Cmhyd1* presented on chromosome VI and the other three on chromosome VII (Figure S6). *Cmhyd4* was separated by over 3000 kb from *Cmhyd3* and *Cmhyd2*, and *Cmhyd3* was separated by over 34 kb from *Cmhyd2* (Figure S6).

### 2.3. Distribution of Hydrophobins in Ascomycota Fungi with Different Lifestyles

**Figure 1:**
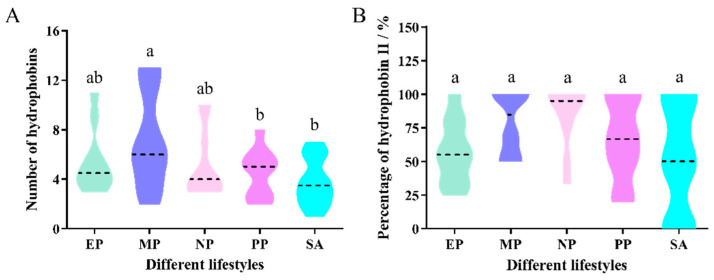
Distribution of hydrophobins in Ascomycota fungi with different lifestyles. (**A**) Number of hydrophobins in Ascomycota fungi with different lifestyles; (**B**) Percentage of class II hydrophobins in Ascomycota fungi with different lifestyles. EP, Entomopathogenic fungi; MP, Mycoparasite; NP, Nematode parasitic fungi; PP, Plant pathogenic fungi; SA, Saprotrophic fungi. The black dotted line represents the median. The different letters over the violin indicate the significant difference at *p* = 0.05.

A survey of the distribution of hydrophobin-encoding genes in ascomycetes revealed a considerable variation in the copy number of hydrophobin genes, ranging from 1 in *Acremonium alcalophilum* to 13 in *T. atroviride* and *T. virens* (Table S2). The average was 5.30 among the tested 47 species, and generally over 70% belonged to class II members. Generally, the number of hydrophobin-encoding genes was the highest in mycoparasite fungi, followed by nematode parasitic, entomopathogenic, and plant pathogenic fungi. Saprotrophic fungi had the fewest hydrophobins, with an average of 3.70 (Figure 1A). The ascomycete fungi with a pathogenic lifestyle tended to be more favored by higher numbers of hydrophobin-encoding genes than the saprotrophic fungi (*p* = 0.049) (Table S2). Among the different pathogenic fungi, the number of hydrophobins from plant pathogenic fungi was fewer than the other fungi (Figure 1A).

The percentage of class II hydrophobin-encoding genes in mycoparasite fungi was the highest (82.05%), followed by nematode parasitic fungi (79.26%), plant pathogenic fungi (70.83%) and entomopathogenic fungi (57.13%), and the lowest was in saprotrophic fungi (56.79%) (Table S2, Figure 1A,B). Some species such as *Tolypocladium paradoxum*, *T. harzianum* and *Clonostachys rosea* only had class II hydrophobins (Table S2).

### 2.4. Phylogeny Analysis Based on the Hydrophobin Proteins of Cordyceps s.l.

**Figure 2:**
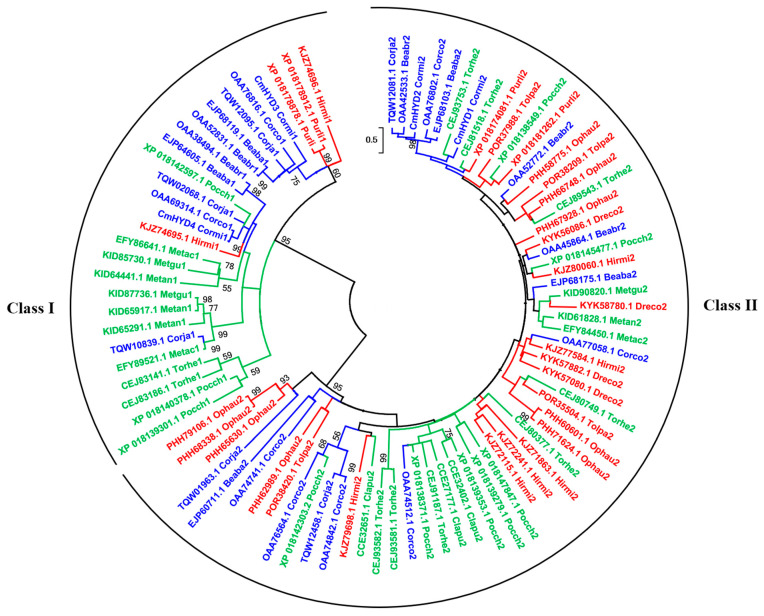
The phylogenetic analysis of hydrophobin proteins of *Cordyceps*. s.l. ML tree showing the phylogenetic relationships between selected fungal hydrophobins using a WAG + G + I model with MEGA X software. The analysis involved 90 hydrophobin protein amino acid sequences from *Cordyceps*. s.l. The tree with the highest log likelihood was −9483.0859. Bootstrap support values above 50% are indicated next to the branches. Three families (*Cordycipitaceae* (**blue**), *Ophiocordycipitaceae* (**red**) and *Clavicipitaceae* (**green**)) are presented in the tree. Fungal species are indicated with the following abbreviations: *Beauveria bassiana*, Beaba; *Beauveria brongniartii*, Beabr; *Claviceps purpurea*, Clapu; *Cordyceps confragosa*, Corco; *Cordyceps javanica*, Corja; *Cordyceps militaris*, Cormi; *Drechmeria coniospora*, Dreco; *Hirsutella minnesotensis*, Hirmi; *Metarhizium acridum*, Metac; *Metarhizium guizhouense*, *Metarhizium anisopliae*, Metan; Metgu; *Ophiocordyceps australis*, Ophau; *Pochonia chlamydosporia*, Pocch; *Purpureocillium lilacinum*, Purli; *Tolypocladium paradoxum*, Tolpa; *Torrubiella hemipterigena*, Torhe.

To analyze the evolutionary relationships of hydrophobins in the species of *Cordyceps* s.l., an Maximum Likelihood (ML) phylogenetic tree was constructed using the amino acid sequences between the first and eighth cysteine residues. A total of 90 hydrophobin proteins, including 28 from *Cordycipitaceae*, 33 from *Ophiocordycipitaceae* and 29 from *Clavicipitaceae*, were assessed (Figure 2, Table S3). The phylogenetic tree strongly supported the two major clades, class I and class II, with relatively high bootstrap values. The bootstrap values of the group of class I were generally higher than those of class II (Figure 2). The number of class II members was obviously higher than class I, which was consistent with the distribution of hydrophobins in Ascomycota (Table S2, Figure 2). CmHYD1 and CmHYD2 were grouped into the clade of class II, while CmHYD3 and CmHYD4 were grouped into the clade of class I, which was consistent with the results of domain structure and hydropathy pattern (Figure 2).

However, in the groups of class I and II, the species did not cluster according to the different families. Class I hydrophobins, CmHYD3 and CmHYD4, and class II hydrophobins, CmHYD1 and CmHYD2, did not group as a cluster, which also occurred in most species. Only a few hydrophobins of the same species grouped as a clade. For example, in *Ophiocordyceps australis,* three of eight class II members grouped as a clade with the high bootstrap (PHH79106.1, PHH68338.1 and PHH65630.1), two (PHH60601.1 and PHH71624.1) grouped as another clade, but the other three scattered in the group of class II. These observations are expected due to the lack of sequence conservation between hydrophobin-encoding genes.

### 2.5. Transcript Patterns of Hydrophobin Genes during the Fruiting Body Development

**Figure 3:**
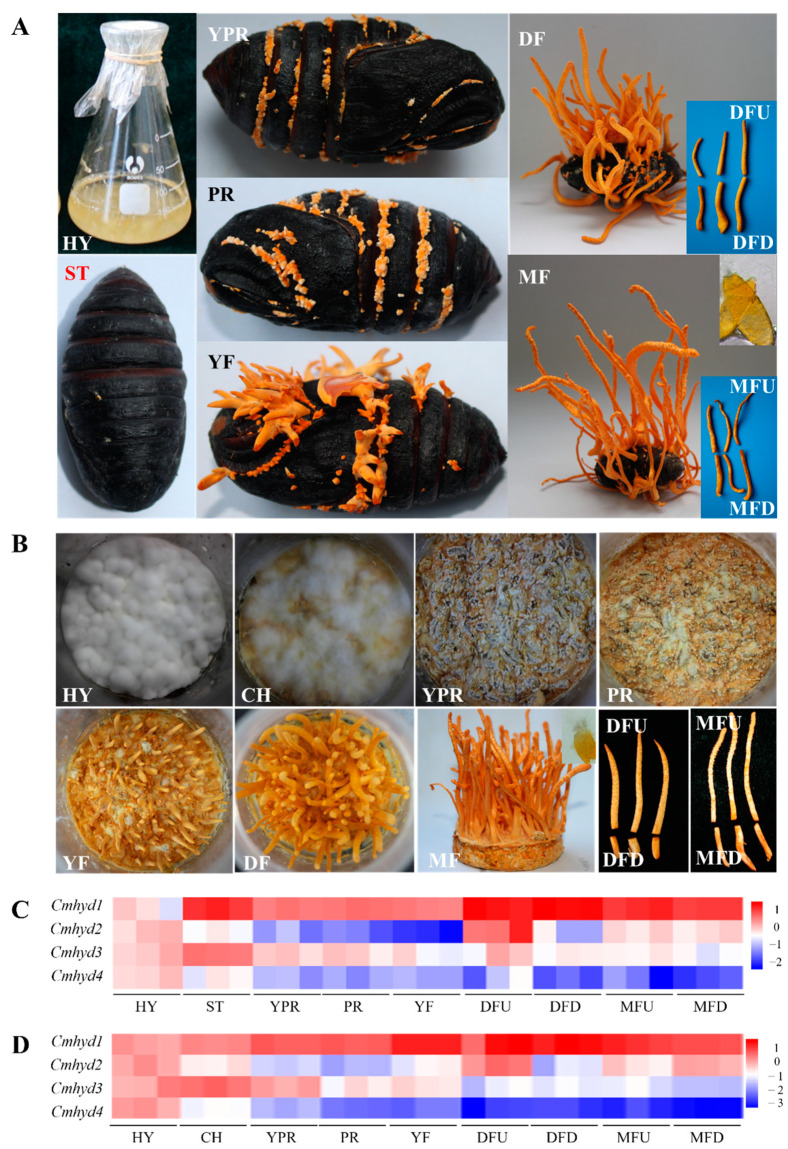
Transcript levels of hydrophobin genes during the fruiting body development. (**A**) Developmental stages of *C. militaris* cultured on *Antheraea pernyi*. HY: the hyphae used for inoculation on *A. pernyi*; ST: sclerotium (mummified pupae) before stroma development; YPR: the mycelium knot tissue before stroma development; PR: sclerotium with initial stroma (stroma < 1 cm); YF: sclerotium with early stage stroma (1 cm < stroma < 2 cm); DF: sclerotium with developed stroma (stroma > 5 cm); MF: fruiting body with mature perithecia, ascus and ascospores. (**B**) Developmental stages of *C. militaris* cultured on wheat media. HY: the hyphae cultured under darkness; CH: the colored hyphae after light irradiation for 4 days; YPR, PR, DF and MF were similar with that cultivated on the insect; MFU: the top fertile part with a superficial visible perithecia; MFD: the sterility stipe. DFU and DFD were the corresponding parts of MF. (**C**) Heatmap of the transcription levels of hydrophobin genes of *C. militaris* cultivated on *A. pernyi*. (**D**) Heatmap of the transcription levels of hydrophobin genes of *C. militaris* cultivated on wheat media. The relative expression levels at the different stages were based on the standard levels of the HY stage in the *A. pernyi* and wheat media, respectively. The different colors of (**C**) and (**D**) represent the log10 of gene transcript folds compared with HY.

The transcripts of four hydrophobin genes were monitored during the growth of fruiting body cultured on *Antheraea pernyi* and wheat medium, respectively. On both conditions, there were two distinct parts of the mature stroma, the sterility stipe (down of mature fruiting body,MFD) and the top fertile part with a superficial visible perithecia (up of mature fruiting body,MFU). DFU (down of developed fruiting body) and DFD (up of developed fruiting body) represented the corresponding parts of MFU and MFD at DF (developed fruiting body) stage (Figure 3A,B). The four hydrophobin genes showed variant transcript patterns (Figure 3C,D), while each hydrophobin gene almost had a similar transcript trend when the fruiting body was cultured on the two different media, *A. pernyi* and wheat. There was a high expression for *Cmhyd1* during the whole fruiting body development. The expression of *Cmhyd1* and *Cmhyd3* increased 13.14-fold and 3.66-fold at the ST (sclerotium) stage compared with the HY (hypha) stage, respectively, when the fruiting body was cultured on *A. pernyi* (Figure 3C, Table S4), indicating that they might play a role in infection and mummification. *Cmhyd2* showed the highest expression in the DFU and low expression in the other stages under both media. *Cmhyd4* showed a very low expression except at the early two stages. When the fruiting body was cultured on wheat medium, the transcript of *Cmhyd3* was up-regulated by 2.41-fold in the CH (colored hyphae) compared with HY cultured under dark conditions, whereas the transcript of *Cmhyd4* was down-regulated significantly after 4 days light treatment (Figure 3D, Table S4). It was indicated that they could response to light stress. The transcript levels of each hydrophobin gene were almost consistent in the two parts of MF (mature fruiting body) stages, MFU and MFD, when the fruiting bodies were cultured on both media. However, there was a significant difference for *Cmhyd2* between DFD and DFU under both media. The transcript level of *Cmhyd2* in DFU was the highest during the whole fruiting body development and was significantly higher than in DFD.

### 2.6. Transcript of Hydrophobin Genes Respond to Light Irradiation

**Figure 4:**
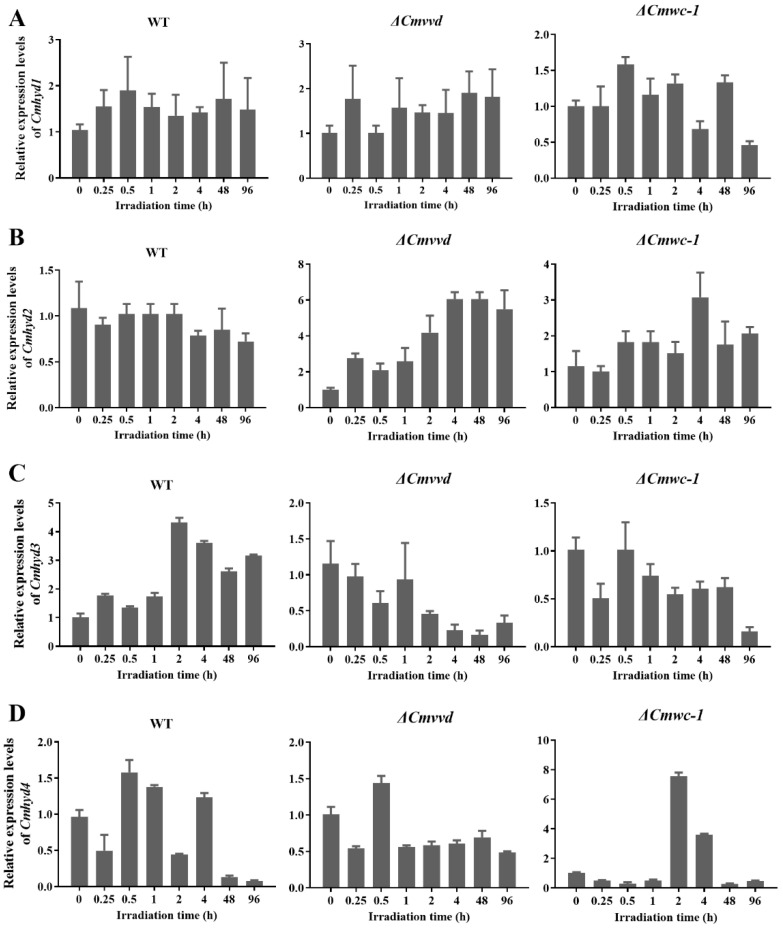
Transcript levels of hydrophobin genes of *Cordyceps militaris* after being exposed to light for different times in wild-type (WT), Δ*Cmvvd* and Δ*Cmwc-1* strains. (**A**) *Cmhyd1*, (**B**) *Cmhyd2*, (**C**) *Cmhyd3*, (**D**) *Cmhyd4*. The relative expression levels at the different light exposure times were based on the standard levels of the light exposure time of 0 (under dark) in the wild-type and deletion mutants, respectively. The average ± standard deviation from three independent measurements with two technical replicates each is shown.

As indicated in Figure 3D, *Cmhyd3* and *4* can respond to light irradiation positively or negatively; therefore, the transcript levels of each hydrophobin gene after light irradiation for different times were compared. In the wild-type strain, *Cmhyd3* showed the highest expression after illumination for 2 h (Figure 4). The transcript of *Cmhyd4* decreased significantly and showed very low expression after light exposure for 48 h and 96 h.

In order to determine whether the light response was related to photoreceptors, the transcript levels of four hydrophobin genes were detected in the photoreceptor gene mutant strains, i.e., Δ*Cmwc-1* and Δ*Cmvvd* strains, after light treatment for 0.25 h to 96 h (Figure 4). There was no light response for *Cmhyd1* in the Δ*Cmwc-1* and Δ*Cmvvd* strains, which was consistent with the wild-type strain (Figure 4A). For *Cmhyd2*, it showed the highest expression after illumination for 4h in the Δ*Cmwc-1* strain, and the transcript level increased gradually with the extension of light treatment time in the Δ*Cmvvd* strain. *Cmhyd3* showed the same trend in the Δ*Cmwc-1* and Δ*Cmvvd* strains so that the light induction disappeared and the transcript level decreased with the extension of light treatment time. The transcript level of *Cmhyd4* increased by 8-fold after light treatment for 2 h in the Δ*Cmwc-1* strain.

### 2.7. Multi-Domain in Hydrophobin Proteins

**Figure 5:**
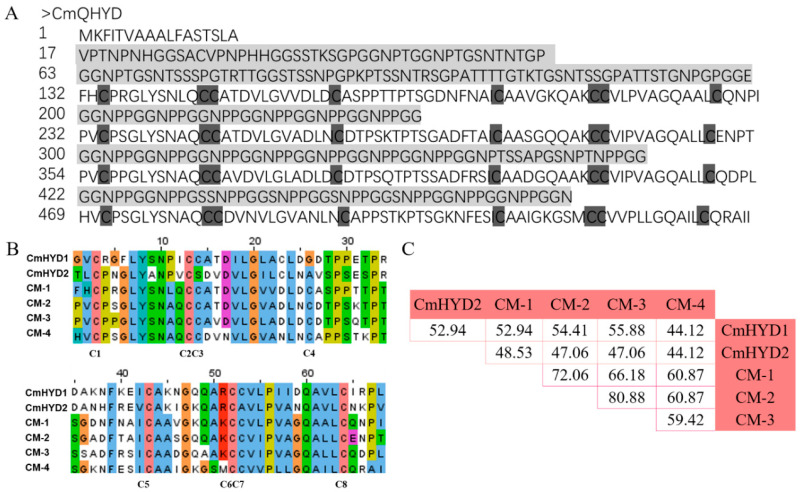
Deduced peptide sequences of CmQHYD. (**A**) The amino terminus was a glycine-rich region and the intervening sequences of four hydrophobin units are highlighted in grey. The conserved cysteine residues of the four hydrophobin domains are shown in black. (**B**) Alignment of the deduced peptide sequences of the four hydrophobin units and two class II hydrophobin proteins, CmHYD1 and CmHYD2. Conserved amino acids are shown in different colors. The alignment was constructed using the Jalview 2.11.0 (SIB Swiss Institute of Bioinformatics, Lausanne, Switzerland) program. CM-1~4 represents the four hydrophobin units of CmQHYD, respectively. (**C**) The percentage of identity between four hydrophobin units of the CmQHYD and CmHYD1 and CmHYD2. The identity referred to the proportion of identical residues at the same site between two sequences.

A quadr-hydrophobin gene (A9K55_003394) was found in the genome of *C. militaris* sequenced by single molecule real-time (SMRT) [36], which presented on the chromosome IV (Figure S6). Unlike the other four *Cmhyd* genes, it was without intron (Figure S1A). The first 17 amino acids represented a signal peptide, according to the SignalP 5.0 analysis, indicating that it was a secreted protein (Figure 5A and Figure S1B). The amino terminus contained a glycine-rich region.

The protein consisted of four units, each showing a significant homology to class II hydrophobins (Pfam06766) (Figure 5A and Figure S1B). The units were separated by GGNPP-repeat regions. The protein was dubbed CmQHYD (*C. militaris* quadr-hydrophobin). Four hydrophobin units had the same cysteine pattern of CX9-CCX11-CX16-CX8-CCX10-C, which was a little different from CmHYD1 and CmHYD2, but corresponded to the consensus defined for the fungal class II hydrophobins (Figure 5, Table S1) [9]. In addition, the hydropathy profiles of the hydrophobin domains of CmQHYD showed significant similarity to class II hydrophobins, as the two cysteine doublets were followed by hydrophobic residues, instead of the hydrophilic residues found in the corresponding regions of class I hydrophobins (Figure S3E).

The homology modelling of the CmQHYD protein showed that each subunit contained one α-helix and four β-hairpins (Figure S4E), like the hydrophobins in *C. militaris*, and the β-hairpins of each subunits conducted a β-barrel (Figure S4E). Each subunit of CmQHYD contained eight cysteine residues that formed four disulfide bridges, which were symmetrically located in almost the same plane in the hydrophobins’ structure: bridge C1-C6, C2-C5, C3-C4 and C7-C8 (Figure S4E). The first bridge, C1-C6, connected the N-terminal loop to the small barrel formed by β-hairpins, the second, C2-C5, connected the α-helix to the β-barrel, and the last two bridges, C3-C4 and C7-C8, were located inside the β-barrel (Figure S4E). Taken together, these data suggested that CmQHYD encoded four modular class II hydrophobins.

A comparison of the four hydrophobin domains of CmQHYD showed that they had an identity of 60.29–80.88% at peptide level. When the four units were aligned with the two class II hydrophobin proteins, CmHYD1 and CmHYD2, a lower identity (<60%) was observed (Figure 5C).

A survey of multi-domain hydrophobin proteins in the published genome of fungi from GenBank revealed that they were only distributed in the fungi of *Cordycipitaceae* and *Clavicipitaceae.* Five species in each family had been found to contain multi-domain hydrophobin proteins, and each species had only one except *B. bassiana* (3) and *T. hemipterigena* (2) (Table 2). These multi-domain hydrophobins had long peptide sequences (264–1187aa) and were 25.91–111.18 kDa in size, N-terminal signal sequences and extracellular location. Although these multi-domain hydrophobins existed in more than one hydrophobin II domain, they were all hydrophilic proteins because of the hydrophilic interval sequences between the hydrophobin II domains (Table 2). There were 2–7 hydrophobin II domains for each multi-domain hydrophobin protein, and one of multi-domain hydrophobins of *T. hemipterigena* (CEJ88606.1) had the most (seven) domains. All the multi-domain hydrophobins were acidic proteins, except that in *P. chlamydosporia* (RZR67753.1).

## 3. Discussion

Hydrophobins are a family of small secreted proteins found exclusively in fungi, and they play various roles in the life cycle of fungi. *C. militaris* is considered as a model organism for the study of over 400 species of *Cordyceps* [29]. In this study, genome wide analysis of the hydrophobin family in *C. militaris* was studied. The distribution of hydrophobins in ascomycete fungi and the evolution of hydrophobins in *Cordyceps* s.l. were also analyzed. It was found that four hydrophobin genes had different responses to light irradiation and different relative transcript levels during the life cycle. The ascomycete fungi with a pathogenic lifestyle had significantly more hydrophobin-encoding genes than saprotrophic fungi. More variability among the class II members was observed than class I, and only a few paralogous proteins were evolved by duplication. Multi-domain hydrophobins were only distributed in the species of *Cordycipitaceae* and *Clavicipitaceae.* The results could be helpful for further function research of hydrophobins in *C. militaris*, and could provide valuable information for the evolution of hydrophobins.

Among the four hydrophobins, two were identified as class I members and the others were class II. According to the alignment of amino acid sequences, three owned conserved eight cysteine residues except that CmHYD2 lacked one cysteine in the two CC doublets, respectively (Figure S2). The homology modelling of CmHYD2 conducted three disulfide bridges (bridge C1-C6, C2-C5, and C4-C8), which were different from the other three hydrophobins with typical four disulfide bridges (Figure S4). Several hydrophobins from *Bjerkandera adusta* and *Phlebia brevispora* had only six out of eight conserved residues, but the missing ones were the first and the sixth residues [37]. Based on a structural analysis, it has been proposed that these two residues formed one of the four disulfide bridges in the hydrophobin molecule [4,38,39]. Therefore, the identified hydrophobins with six conserved cysteine residues retained the biological activity, in that their structure would still be stabilized by three disulfide bridges [37]. In addition, the spacing between cysteines of CmHYD2 was consistent with the conserved cysteine pattern of class II (Table S1) [35] as well as the hydropathy plot and hydrophobicity (Figures S2 and S3; Table 1). CmHYD2 should be a hydrophobin class II member.

The location of the genes encoding four hydrophobins and one quadr-hydrophobin on the chromosomes were firstly analyzed (Figure S6). *Cmhyd1* and *Cmqhyd* presented on chromosomes VI and IV, respectively. The strength of linkage between two genes depended upon the distance between the genes on the chromosome [40]. Though the other three hydrophobin genes were all on chromosome VII, *Cmhyd4* was separated by a long distance from *Cmhyd3* and *Cmhyd2.* They are obviously unlinked. Whether *Cmhyd3* and *Cmhyd2* are linked together should be verified by the construction of linkage maps.

Though there was only class I hydrophobin, a significant expansion of hydrophobin-encoding genes in basidiomycetes was reported (1–40 copies) [8]. In the present study, it was found that the copy number of hydrophobin genes in ascomycetes ranged from 1 to 13 (Table S2) with an average of 5.30, which confirmed the contraction of hydrophobins in ascomycetes. It was reported that the non-pathogenic fungi had more hydrophobins compared with the pathogenic fungi [8]. However, 47 ascomycetes species with different lifestyles were analyzed, and it was found that the number of hydrophobin-encoding genes in the fungi with a pathogenic lifestyle was significantly higher than the saprotrophic fungi. This inconsistency was explained by the fact that the species used in this study were confined within Ascomycota, but species of basidiomycetes with abundant hydrophobins and more non-pathogenic species were included in the study of Mgbeahuruike et al. [8]. In ascomycetes, the number of hydrophobins as well as the class II hydrophobin-encoding gene was the highest in mycoparasite fungi, indicating that hydrophobins may be involved in fungal antagonistic interactions [8,41], and mycoparasite fungi would need more hydrophobins to aggress their fungal food, which also arms themselves with hydrophobins.

Phylogenetic analysis of hydrophobins from both basidiomycetes and ascomycetes [2,8], a representative set of basidiomycetes and ascomycetes, respectively, [8,35,37] has been performed to clarify the evolution of hydrophobins. In this study, phylogenetic analysis was performed based only on the hydrophobin proteins of *Cordyceps* s.l. The amino acid sequences from C1 to C8 were aligned because of the poor amino acid sequence conservation of the hydrophobins. The result strongly supported the two major clades, class I and class II, with relatively high bootstrap values, which was consistent with the previous report [8]. However, the bootstrap values of class I were generally higher than those of class II in the ML phylogenetic tree (Figure 2), indicating higher variability among the class II members. It was reported that in the phylogenetic analysis from both basidiomycetes and ascomycetes, class I hydrophobins had more sequence variation than the class II hydrophobins [2] because class I hydrophobins were observed in both basidiomycetes and ascomycetes. Another obvious feature from the phylogenetic analysis was the existence of a few paralogous proteins. Examples for this were: XP_018178878.1 and XP_018178912.1 from *Purpureocillium lilacinum*; PHH79106.1, PHH68338.1 and PHH65630.1 from *O. australis*; PHH60601.1 and PHH71624.1 from *O. australis*; CEJ93582.1 and CEJ93581.1 from *Torrubiella hemipterigena*. Most of these twins formed a terminal branch, or were connected by a single node, indicating that they were aroused by gene duplication. However, CmHYD3 and CmHYD4, and CmHYD1 and CmHYD2 were not produced by gene duplication. Both birth-and-death evolution [35] and convergent evolution [42] have been proposed for hydophobin proteins. More data were needed to resolve the question of ancestry of the two classes of hydrophobins.

Different transcript patterns of 4 hydrophobins during the life cycle of *C. militaris* indicated there may be different functions. *Cmhyd1*, one of the class II members, showed a high expression compared with HY stage during the whole life cycle cultured on both insect and wheat media (Figure 3C,D), implying that *Cmhyd1* was not only important for fruiting body development, but also played a role during the infection on the insects and mummification. It was reported that the absence of class II proteins in basidiomycetes may indicate that only the class I proteins was important for fruiting body development [8]. However, in this study, class I hydrophobins, both *Cmhyd3* and *Cmhyd4* showed a very low expression in all the fruiting body development stages. The other class II hydrophobin member, *Cmhyd2* showed the highest expression only in the DFU and low expression in the other stages under both media. An upregulation 3.66-fold was observed at ST stage for *Cmhyd3* when the fruiting body was cultured on *A. pernyi* (Figure 3C), indicating that *Cmhyd3* may play a role in the insect virulence, which was consistent with the report of class I hydrophobin-encoding gene in *B. bassiana* [15]. *Cmhyd4* expressed highest in HY stage under dark conditions, indicating it was regulated by light signal negatively. These diversity of transcript patterns and their putative functions would add the complexity of evolution of class I and II hydrophobins.

The transcripts of some class I and II hydrophobin-encoding genes were stimulated by light in some fungi, including *N. crassa* [25], *T. atroviride* [23,24] and *T. reesei* [21], and this stimulation has also been linked to the circadian clock [22]. In *C. militaris*, *Cmhyd1* did not respond to light irradiation, whereas the other three had different light-responses. The transcript of *Cmhyd2* had no response in the wild-type strain but the transcript level increased in the two photoreceptor mutant strains, *ΔCmwc-1* and *ΔCmvvd* (Figure 4), which was consistent with *hyd2* in *S. commune* which responded to light in the *Δwc2Δwc2* dikaryon compared with the wild-type dikaryon [20]. The light induction of *Cmhyd3* in the wild-type strain disappeared in both *ΔCmwc-1* and *ΔCmvvd* strains, indicating that its light response was dependent on the photo receptors *Cmwc-1* and *Cmvvd.* The transcript of *Cmhyd4* decreased significantly and showed very low expression after light exposure for 48h and 96h in the wild-type strain and increased by 8-fold after light treatment for 2h in the *ΔCmwc-1* strain, indicating that it was regulated by light signals and CmWC-1 negatively. The functions of four hydrophobins and the relation with photoreceptors are now being explored by gene deletion and protein–protein interactions in our laboratory.

A hydrophobin with four hydrophobin II domains (Pfam06766), CmQHYD, was found during the search for hydrophobins in the genome of *C. militaris* [36]. The multi-domain hydrophobin protein was first reported in *Claviceps fusiformis*, dubbed *cfth1* [43,44]. There were four hydrophilic GN-rich (G: glycine, N: asparagine) stretches preceding each hydrophobin domain in CmQHYD, which was like CFTH1, but CmQHYD did not contain the DYP (Asp-Tyr-Pro residues) motif which was presented in both GN-spacers in CFTH1. The identity (60.29–80.88%) of the four hydrophobin domains of CmQHYD were higher at peptide level, implying that these internal domains may more likely stem from a common ancestor (Figure 5C). It was also proposed that the occurrence of the *Claviceps* multipartite hydrophobins would be due to multiplication of some of the class II hydrophobins by tandem duplication [35].

A survey of multi-domain hydrophobin proteins in the published genome of fungi from GenBank revealed that they were only distributed in the species of *Cordycipitaceae* and *Clavicipitaceae* (Table 2). The multi-domain hydrophobin CPPH1 in *C. purpurea* did not participate in the morphology, growth rate, sporulation, or hydrophobicity of spores or virulence on rye [45]. It is necessary to further study the CmQHYD functions in *C. militaris*.

## 4. Materials and Methods

### 4.1. Strains, Media and Growth Conditions

The *C. militaris* strain 453 (CGMCC 3.16323) and disruption strains of photoreceptor *Cmwc-1* (*ΔCmwc-1*, [31]) and *Cmvvd* (*ΔCmvvd*, [34]) were maintained on potato dextrose agar (PDA) at 20 °C with appropriate antibiotics for the mutant strains. Liquid cultures were performed in media with 200 g/L potato, 20 g/L glucose, 3 g/L peptone, 1 g/L KH_2_PO_4_, and 0.5 g/L MgSO_4_.

### 4.2. Sequence Search and Annotation of Hydrophobin Genes in Cordyceps militaris

Two sequenced *C. militaris* genomes (Accession: SRA047932 and PRJNA323705) [36,46] were used to search for hydrophobins. The sequences were examined for domains using Pfam to verify their function as hydrophobins [47]. The noted hydrophobin protein sequences were then used to align with *C. militaris* genomes by Basic Local Alignment Search Tool (BLAST) to ensure finding all hydrophobin members [48]. The presence and location of signal peptide cleavage sites were obtained using SignalP 5.0 [49]. Hydrophobicity profiles were obtained at the ExPASy server using the Kyte and Doolittle scale with default parameters [50].

### 4.3. Homology Modeling of Hydrophobins

Homology modeling of hydrophobins including CmHYD1–CmHYD4 and CmQHYD was constructed by the SWISS-MODEL server (https://swissmodel.expasy.org/) (SIB Swiss Institute of Bioinformatics, Lausanne, Switzerland). Firstly, the sequence of hydrophobins was submitted to the SWISS-MODEL server to determine the optimal template for model building. Then, we ranked the top 10 matches according to their sequence identity and QMEAN4 scores. The optimal templates were selected to build alternative models for hydrophobins. The identical network of disulfide bridges was added with PyMOL software [51].

### 4.4. Distribution of Hydrophobins in Ascomycota Fungi with Different Life Styles

Hydrophobin-encoding genes were searched from the open genome of 47 Ascomycetes species following a protocol by Rineau et al. [52]. Briefly, reference hydrophobins from the genomes were first retrieved. For each of the genomes, the following procedures were used: (1) Each of these reference hydrophobin sequences were blasted against each of the genomes (filtered gene models database, BlastP and tBlastn, threshold e-value = 10^−5^), (2) All the proteins predicted to contain Pfam06766 and Pfam01185 were retrieved, (3) All protein sequences bearing the hydrophobin type I (C-X_5–7_-C-C-X_19–39_-C-X_8–23_-C-X_5_-C-C-X_6–18_-C-X_2–13_) or type II (C-X_9_-CC-X_11_-C-X_14–16_-C-X_8_-C-C-X_10_-C-X_6–7_) signature sequences were retrieved [37]. Then, all the sequences retrieved through at least one of the three methods were re-blasted (tBlastn, threshold e-value =1 × 10^−5^) against each genome. A total of 243 hydrophobins were obtained. The classification was checked by Pfam carefully. These ascomycetes species were subdivided as entomopathogenic, mycoparasite, nematode parasitic, plant pathogenic and saprotrophic lifestyles. The data were analyzed by one-way analysis of variance (ANOVA). Significant differences were determined by Duncan’s multiple range tests. Data analyses were completed with SPSS 19.0 (SPSS, Inc., Chicago, IL, USA) and GraphPad Prism 8.

### 4.5. Phylogenetic Analysis of Hydrophobins in Cordyceps Sensu Lato

Hydrophobin proteins of *Cordyceps* sensu lato species including *Cordycipitaceae*, *Ophiocordycipitaceae* and *Clavicipitaceae* and, in total, 90 amino acid sequences were downloaded from GenBank (Table S3), and the classification of hydrophobins was carefully checked by Pfam analysis. The amino acid sequences between the first and the eighth cysteine residues of four *C. militaris* hydrophobins and those downloaded hydrophobins were aligned using the MAFFT program version 7 with default parameters [53,54], and then manually refined. Unambiguously aligned positions were used for constructing phylogenetic trees with Maximum Likelihood (ML) using MEGA X [55]. The optimal model of evolution was determined as WAG + G + I using the Find Best Protein Model provided by MEGA X [55]. Then, the ML tree was constructed (gap treatment: Use all sites) based on the Nearest-Neighbor-Interchange (NNI) method with MEGA X. Consistency of the phylogenetic estimate was evaluated with the ultrafast bootstraps method test for 1000 replications.

### 4.6. Transcript Analysis of Hydrophobin Genes during the Fruiting Body Development

The *C. militaris* fruiting bodies were cultivated on host silkworm *Antheraea pernyi* and wheat medium according to the method of our previous report [56]. Samples of different developmental stages were harvested. When the fruiting bodies were cultured on *A. pernyi,* the hyphae, which were cultured on potato dextrose broth in the dark for 2d and used as inoculum, were designated as hyphae (HY). The mummified pupae covered with mycelia before stroma development were designated as the sclerotium (ST). The mycelium knot tissue before stroma development was named as young primordium (YPR). The samples of stroma with lengths <1 cm, 1–2 cm and 5 cm were designated as the primordium (PR), young fruiting body (YF) and developed fruiting body (DF), respectively. The fruiting body with mature perithecia and ascospores was designated as the mature fruiting body (MF).

When the fruiting body was cultivated on wheat medium, the hyphae covering the medium, which were cultured in the dark, were designated as hyphae (HY). Then, the hyphae were exposed under light irradiation. After being cultured under 12 h dark:12 h light for 4 days, the hyphae turned orange and were designated as colored hyphae (CH). The samples of YPR, PR, YF, DF, and MF were similar with those cultivated on insects.

RNA extraction and RT-qPCR assay were performed following the description before [34]. The primers used in RT-qPCR analysis are listed in Table S5. The *rpb1* gene (CCM_05485) was used as an internal standard [57]. The 2^−ΔΔCt^ method was used to calculate the relative expression levels [58]. The obtained data represented three biological replicates, with two technical replicates each. The heatmaps of the transcription levels were drawn by Matrix2png (https://matrix2png.msl.ubc.ca/) (Columbia Genome Center, Columbia University, New York, NY, USA).

### 4.7. Transcript Analysis of Hydrophobin Genes after Light Exposure for Different Times

Wild type, *ΔCmwc-1* [31] and *ΔCmvvd* [34] strains were cultured on PPDA (10g/L peptone was added in the PDA medium) under constant dark at 20 °C for 20 days before being transferred to constant light (1000 lux) at 20 °C. The samples were harvested after being exposed for 0.25–96 h. RNA extraction and RT-qPCR were performed as above. The graphs were drawn by GraphPad Prism 8 (Graphpad Software, Inc. La Jolla, CA, USA).

## 5. Conclusions

A total of four hydrophobin-encoding genes were identified and characterized in *C. militaris*. Each hydrophobin gene had a different transcript pattern during the life cycle and different responses to light irradiation. The ascomycete fungi with a pathogenic lifestyle tended to be more favored by higher numbers of hydrophobin-encoding genes than the saprotrophic fungi. There was more variability among the class II members than class I, and only a few paralogous proteins were evolved by duplication. Multi-domain hydrophobins were only distributed in the species of *Cordycipitaceae* and *Clavicipitaceae,* whose function remains to be clarified.

## Figures and Tables

**Table 1:** Hydrophobins identified in the genome of *Cordyceps militaris.*

Name	Protein ID *	Chr. **	Genomic Location ***	AA ****	MW ***** (kDa)	GRAVY ******	Class	Signal Peptide
*Cmhyd1*	CCM_03537/ A9K55_005453	6	356807–357251	105	10.57	0.37	II	Yes
*Cmhyd2*	CCM_06854/ A9K55_008053	7	3749538–3749938	99	10.42	0.412	II	Yes
*Cmhyd3*	CCM_06862/ A9K55_008043	7	3715724–3716248	138	13.48	0.583	Ι	Yes
*Cmhyd4*	CCM_07964/ A9K55_007066	7	398330–398703	104	10.35	0.478	Ι	Yes

* Protein ID indicates the ID in the *C. militaris* genomes SRA047932 and PRJNA323705, respectively. ** Chr. indicates chromosome. *** Genomic location indicates the location at the corresponding chromosome. **** indicates the number of amino acids. ***** indicates the molecular weight of protein. ****** GRAVY indicates the grand average of hydropathicity.

**Table 2:** The multi-domain hydrophobin proteins found in fungi of *Cordycipitaceae* and *Clavicipitaceae.*

Species	No. MH *	GenBank ID	No. H II ** Domain	AA	MW (kDa)	GRAVY ***	pI ****
*Cordycipitaceae*							
*Beauveria bassiana*	3	PMB64239.1	4	734	71.74	−0.48	6.1
		PQK10896.1	3	593	57.46	−0.50	6.01
		KAF1736859.1	3	596	58.22	−0.54	6.59
*Cordyceps militaris*	1	ATY58761.1	4	537	51.00	−0.36	6.16
*Cordyceps fumosorosea*	1	XP_018708370.1	5	599	58.36	−0.31	5.01
*Cordyceps javanica*	1	TQV96986.1	2	264	25.91	−0.16	5.15
*Cordyceps confragosa*	1	OAA82275.1	4	605	57.51	−0.44	5.77
*Clavicipitaceae*							
*Torrubiella hemipterigena*	2	CEJ88606.1	7	1187	111.18	−0.58	4.06
		CEJ95278.1	3	719	67.70	−0.53	4.23
*Claviceps fusiformis*	1	CAB61236.1	3	394	36.81	−0.53	4.70
*Claviceps purpurea*	1	CAD10781.1	5	756	69.90	−0.73	5.27
*Moelleriella libera*	1	KZZ89374.1	4	508	49.50	−0.59	4.74
*Pochonia chlamydosporia*	1	RZR67753.1	3	402	38.21	−0.62	7.67

* MH indicates multi-domain hydrophobin. ** HII indicates hydrophobin II. *** GRAVY indicates grand average of hydropathicity. **** indicates isoelectric point.

## Data Availability

The data presented in this study are available in the manuscript and supplementary material.

## Supplementary Materials

Supplementary Materials can be found at https://www.mdpi.com/1422-0067/22/2/643/s1.
