# Supplementary material for: Cysteine-Rich Hydrophobin Gene Family: Genome Wide Analysis, Phylogeny and Transcript Profiling in Cordyceps militaris
Source: Int J Mol Sci. 2021 Jan 11;22(2):643. doi: 10.3390/ijms22020643 (PMC7827705; doi:10.3390/ijms22020643)
Supplement: Supplementary file 1 [file ijms-22-00643-s001.zip › ijms-987317-supplementary/Online Resource 2.docx]

**Cysteine-rich hydrophobin gene family: Genome wide analysis, phylogeny and transcript profiling in *Cordyceps militaris***

Xiao Li^a, b^, Fen Wang^a^, Yanyan Xu^a^, Guijun Liu^c^, Caihong Dong^a, d*^

^a^State Key Laboratory of Mycology, Institute of Microbiology, Chinese Academy of Sciences, Beijing, China; ^b^University of Chinese Academy of Sciences, Beijing 100039, China; ^c^Beijing Radiation Center, Beijing, China; ^d^Guizhou Key Laboratory of Edible Fungi Breeding, Guizhou Academy of Agricultural Sciences, Guiyang, China

* Corresponding author

Caihong Dong

State Key Laboratory of Mycology

Institute of Microbiology, Chinese Academy of Sciences

NO.3 Park 1, Beichen West Road, Chaoyang District, Beijing, 100101 China

E-mail: [dongch@im.ac.cn](mailto:dongch@im.ac.cn)


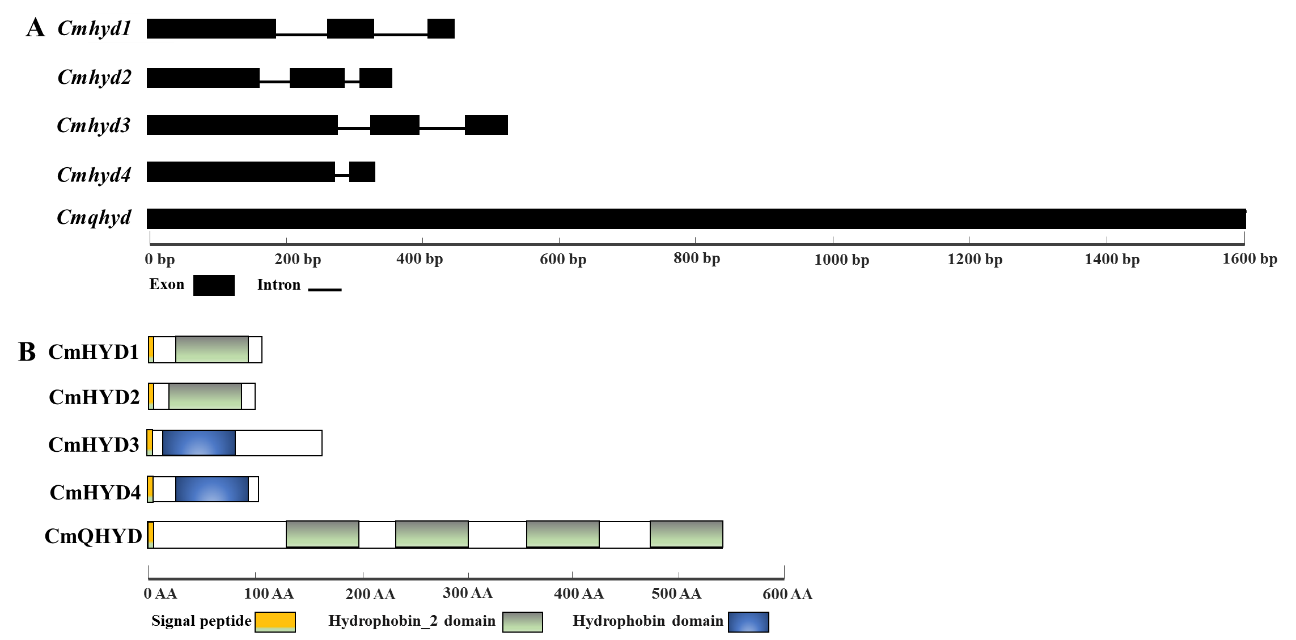
**Figure S1**. The gene structure and function domain of hydrophobins in *Cordyceps militaris*.

**(A)** Gene structure; **(B)** Function domain.


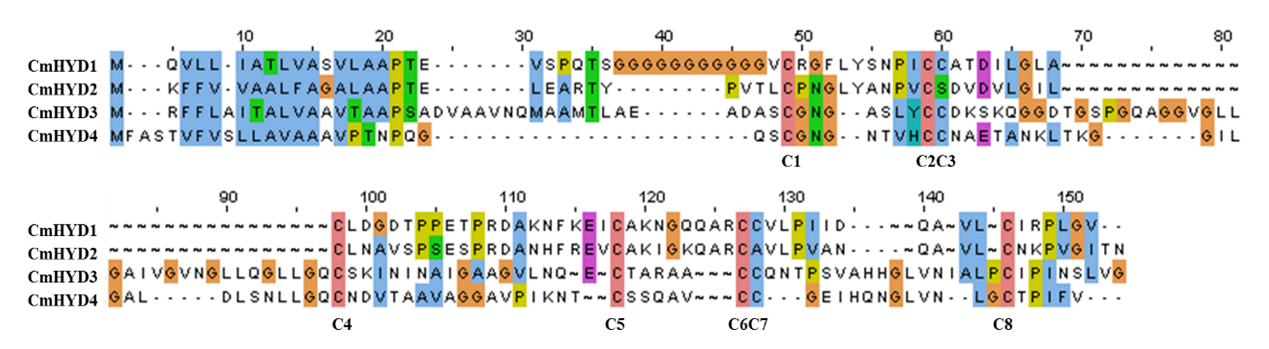
**Figure S2**. Sequence alignment of hydrophobins from *Cordyceps militaris*.

Conserved amino acids were shown in different colors.


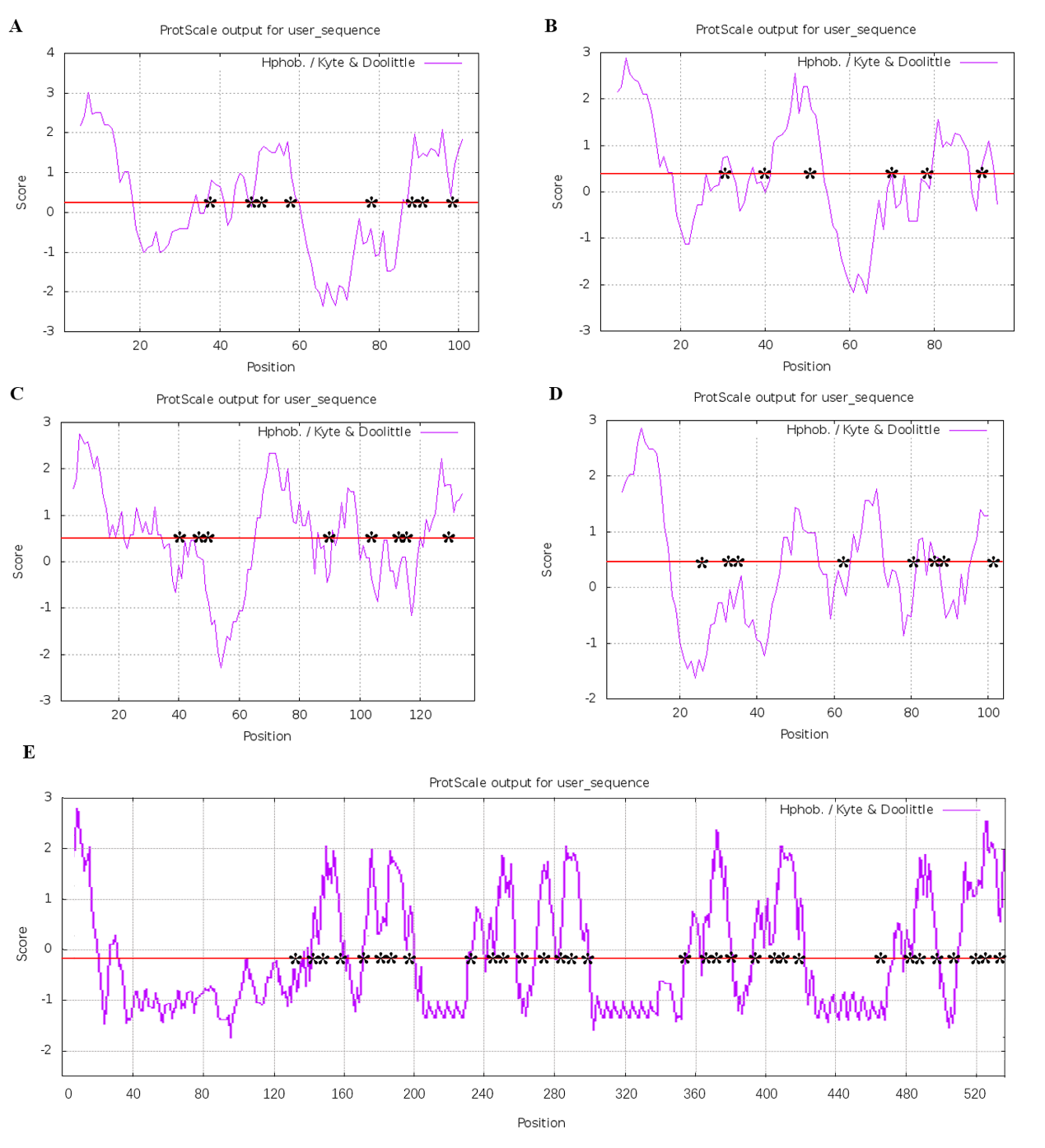
**Figure S3**. Hydropathy plots of hydrophobins in *Cordyceps militaris*.

**(A)** CmHYD1, **(B)** CmHYD2, **(C)** CmHYD3, **(D)** CmHYD4, **(E)** CmQHYD.

Hydropathy plot was predicted with ProtScale at the ExPASy server using the [Kyte & Doolittle](https://web.expasy.org/protscale/pscale/Hphob.Doolittle.html) scale with default parameters (https://web.expasy.org/protscale/). Hydrophobic amino acids showed more positive peaks whereas hydrophilic amino acids showed more negative peaks. Asterisks indicated the conserved cysteine residues positions. The red line represented the average hydropathicity.


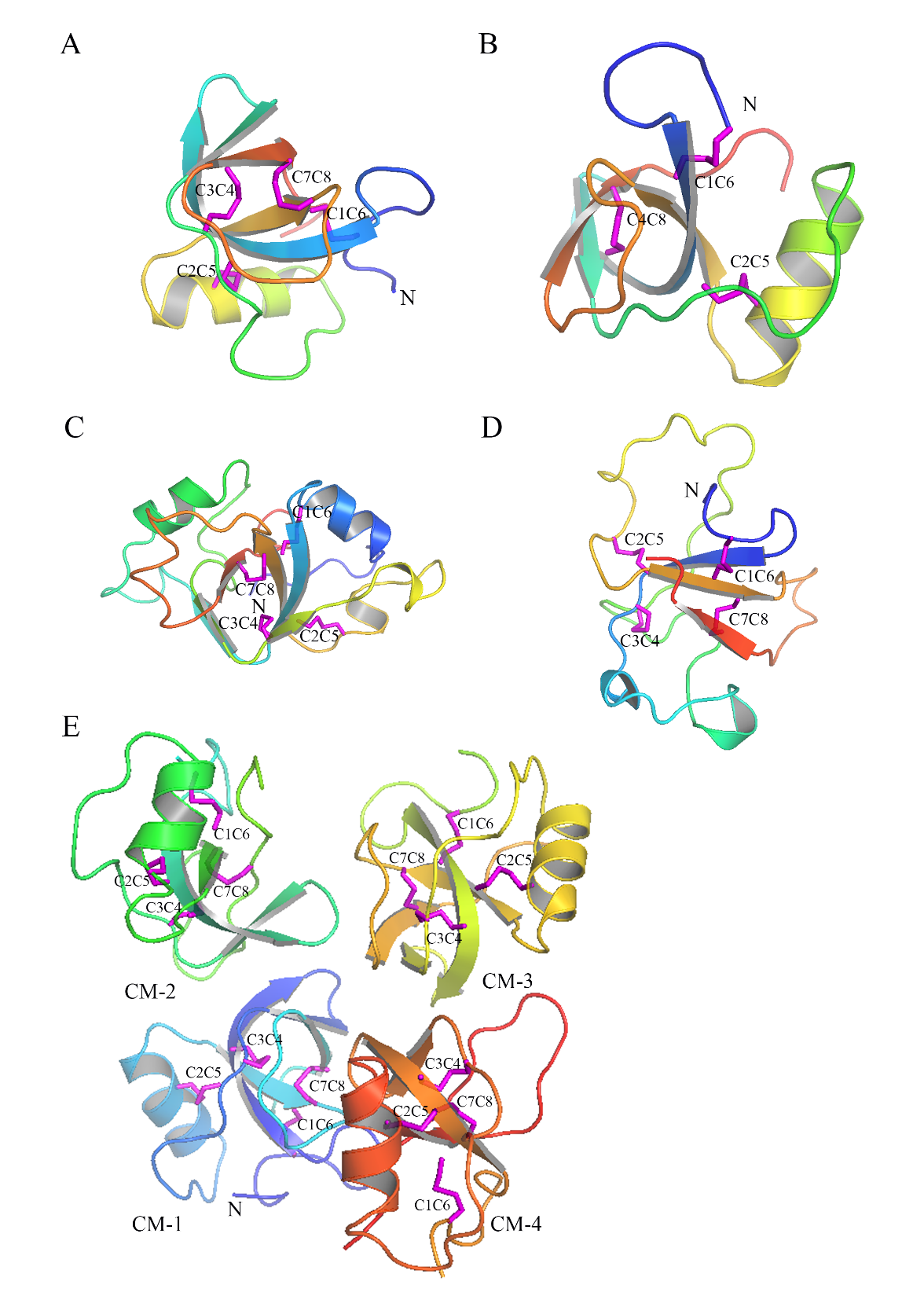
**Figure S4**. Homology modeling of four hydrophobins and quadr-hydrophobin

**(A)** CmHYD1, **(B)** CmHYD2, **(C)** CmHYD3, **(D)** CmHYD4, **(E)** CmQHYD.

The disulfide bridges Cys1-Cys6, Cys2-Cys5, Cys3-Cys4, and Cys7-Cys8 in CmHYD1, CmHYD3, CmHYD4 and CmQHYD. Cys1-Cys6, Cys2-Cys5 and Cys4-Cys8 in CmHYD2 were shown. The identical networks of disulfide bridges (magentas) were added with PyMOL software. CM-1~4 represented the four hydrophobin units of CmQHYD, respectively.

**Figure S5**. The model sequences analysis of *Cordyceps militaris* hydrophobins homology modeling. A. CmHYD1; B. CmHYD2; C. CmHYD3; D. CmHYD4; E. CmQHYD.

**(A)** CmHYD1

>pdb|2FZ6|C Chain C, Hydrophobin-1, *Trichoderma reesei*

SNGNGNVCPPGLFSNPQCCATQVLGLIGLDCKVPSQNVYDGTDFRNVCAKTGAQPLCCVAPVAGQALLCQTAVGA


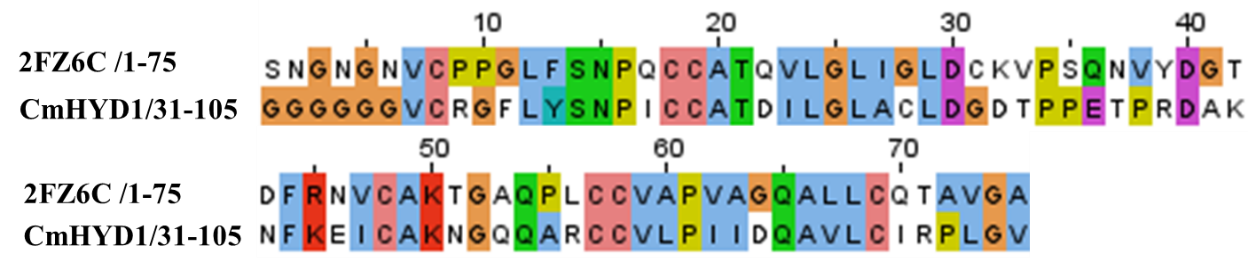
Seq Identity: 45.83%

**(B)** CmHYD2

>pdb|2FZ6|C Chain C, Hydrophobin-1, *Trichoderma reesei*

SNGNGNVCPPGLFSNPQCCATQVLGLIGLDCKVPSQNVYDGTDFRNVCAKTGAQPLCCVAPVAGQALLCQTAVGA


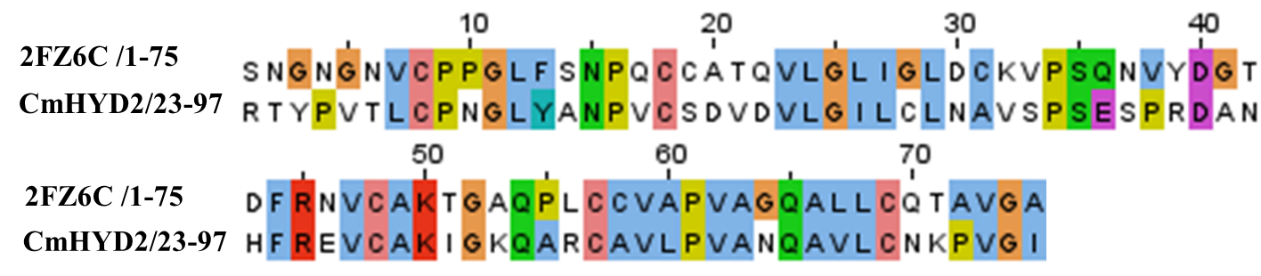
Seq Identity: 48.53%

**(C)** CmHYD3

>pdb|6GCJ|A Chain A, Hydrophobin, *Aspergillus fumigatus* A1163

SLPQHDVNAAGNGVGNKGNANVRFPVPDDITVKQATEKCGDQAQLSCCNKATYAGDVTDIDEGILAGTLKNLIGGGSGTEGLGLFNQCSKLDLQIPIIGIPIQDLVNQKCKQNIACCQNSPSDASGSLIGLGLPCIALGSIL

Seq Identity: 33.06%


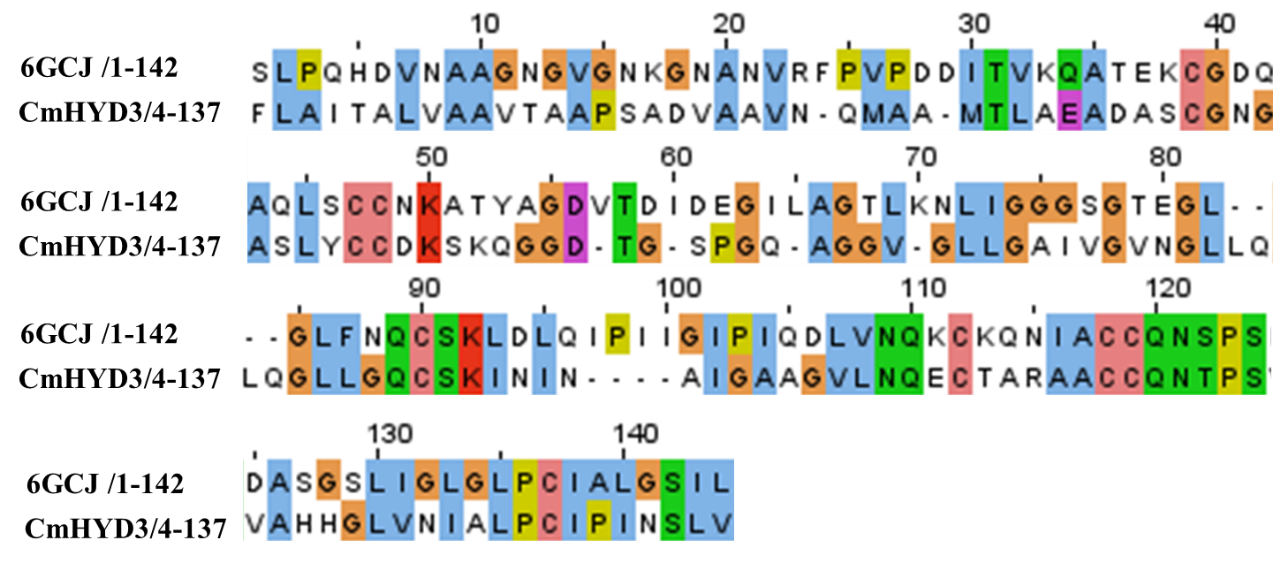


**(D)** CmHYD4

>pdb|2N4O|A Chain A, Hydrophobin-like protein MPG1, *Pyricularia oryzae* 70-15


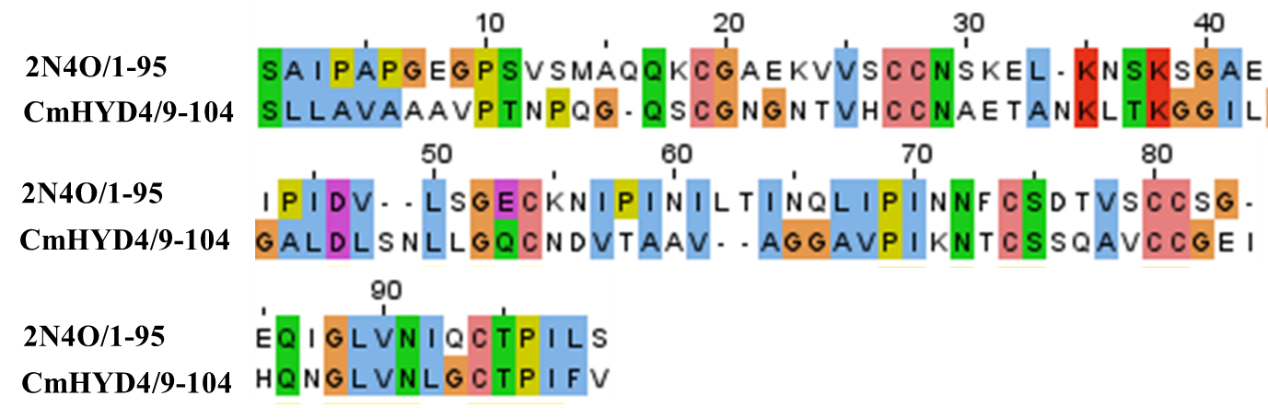
SAIPAPGEGPSVSMAQQKCGAEKVVSCCNSKELKNSKSGAEIPIDVLSGECKNIPINILTINQLIPINNFCSDTVSCCSGEQIGLVNIQCTPILS

Seq Identity: 34.67%

**(E)** CmQHYD

>pdb|2FZ6|A Chain A, Hydrophobin-1, *Trichoderma reesei*

SNGNGNVCPPGLFSNPQCCATQVLGLIGLDCKVPSQNVYDGTDFRNVCAKTGAQPLCCVAPVAGQALLCQTAVGA


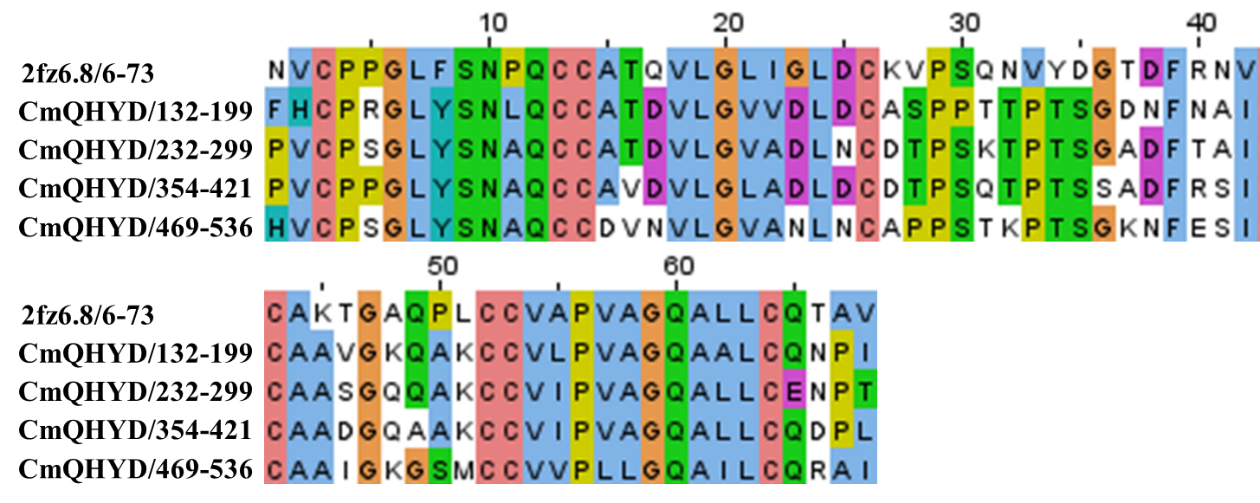
Seq Identity: 33.06%

**
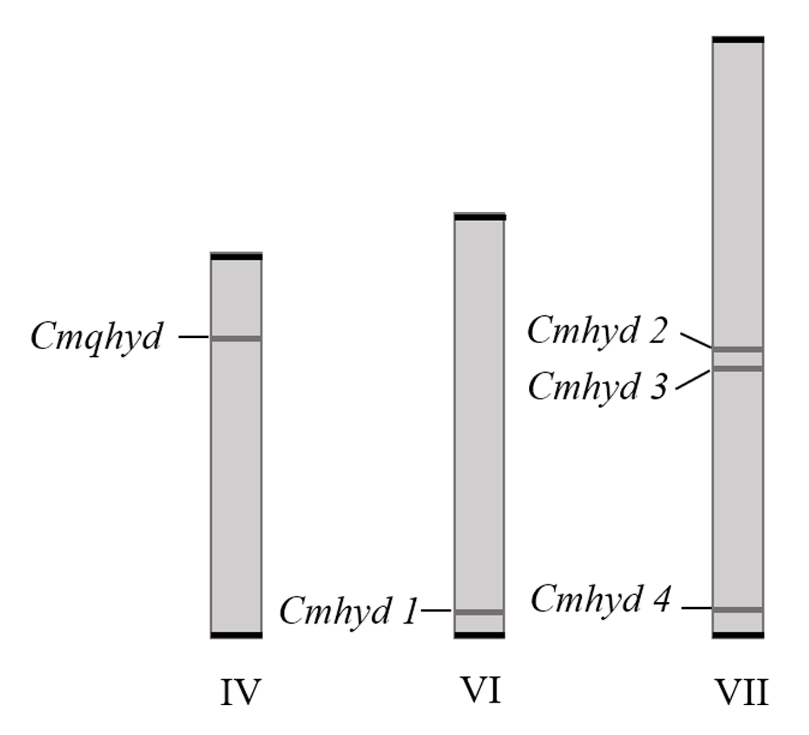
Figure S6**. Chromosomal location of hydrophobin genes in *Cordyceps militaris*.
